# Radiomic Analysis of Craniopharyngioma and Meningioma in the Sellar/Parasellar Area with MR Images Features and Texture Features: A Feasible Study

**DOI:** 10.1155/2020/4837156

**Published:** 2020-02-18

**Authors:** Zerong Tian, Chaoyue Chen, Yang Zhang, Yimeng Fan, Ridong Feng, Jianguo Xu

**Affiliations:** ^1^West China School of Medicine, West China Hospital, Sichuan University, Chengdu 610041, China; ^2^Department of Neurosurgery, West China Hospital, Sichuan University, Chengdu 610041, China; ^3^Department of Ophthalmology, West China Hospital, Sichuan University, Chengdu 610041, China; ^4^State Key Laboratory of Biotherapy and Cancer Center, West China Hospital, Sichuan University, Chengdu 610041, China

## Abstract

**Purpose:**

To investigate the ability of qualitative Magnetic Resonance (MR) images features and quantitative Magnetic Resonance Imaging (MRI) texture features in the contrastive analysis between craniopharyngioma and meningioma.

**Method:**

A total number of 127 patients were included in this study (craniopharyngioma = 63; meningioma = 64). All the features analyzed in this study were acquired from preoperative MRI images. Qualitative MR images features were evaluated with chi-square tests or Fisher exact test, while MRI texture features were evaluated with the Mann–Whitney *U* test with the Benjamini–Hochberg method. Then binary logistic regression analysis for texture features was performed to evaluate their ability as independent predictors, and the diagnostic accuracy was calculated next for these texture features with high abilities as independent predictors using receiver operating characteristic (ROC) curves.

**Results:**

Four qualitative MR images features showed significant difference between craniopharyngioma and meningioma, but only cystic alteration could be considered as diagnostic independent predictors. Meanwhile, three quantitative parameters, histogram-based matrix- (HISTO-) Skewness, Grey-level co-occurrence matrix- (GLCM-) Contrast on contrast-enhanced images, and HISTO-Skewness on images of T2-weighted imaging (T2WI), showed promising abilities in the contrastive analysis. Besides, these texture features were found significantly to be relative to cystic alteration.

**Conclusion:**

MR images features and texture features were useful in the contrastive analysis of craniopharyngioma and meningioma. Furthermore, qualitative MR images features and MRI texture features could be related to each other.

## 1. Introduction

Craniopharyngioma and meningioma are two of the most common benign tumors in the sellar or parasellar area. Craniopharyngioma presents approximately 2.5%–4% of the brain tumor. It can be detected at any age; besides, it is the overwhelming major tumor (approximately 90%) of the pituitary region neoplasms in children [1–4]. Meningioma presents approximately 36% of all central nervous system tumors, with an occurrence rate of 7.61/100000 [5, 6]. The patients with craniopharyngioma and meningioma in the sellar/parasellar area may suffer from similar symptoms, headache, visual change, and pituitary dysfunction, which are caused by the anatomical proximity of the tumor to the optic nerve/chiasma and hypothalamic-pituitary axes [7–10]. Magnetic resonance imaging (MRI) is the standard preoperative modality to detect craniopharyngioma or meningioma. MRI can morphologically assess the size, anatomic location, and proximal structure of the tumor and possible histopathologic changes. The different MRI imaging features can provide feasible information in the contrastive analysis [11]. It is crucial to distinguish meningioma in th sellar/parasellar area from craniopharyngioma because of the differences in treatment recommendation and prognosis. However, the contrastive analysis of these tumors still remains to be a challenge because craniopharyngiomas may mimic meningiomas in the sellar/parasellar area in some cases [12].

Texture analysis (TA) is a method to describe the voxel-value frequency distribution and the spatial organization of voxel value, which can reflect how each voxel value differs from the neighbor voxel values. During the analysis process, several matrices are used to capture information from clinical images, and each matrix enables the calculation of several heterogeneity descriptors [13]. Texture analysis has been widely used in detection and classification of various tumors, like tumors in the brain, lung, breast, and prostate [14–17]. Previous studies identified the values of TA in grading meningiomas; however, the values in diagnosis are unknown [17]. Besides, TA has never been performed in craniopharyngioma. In this study, we analyzed Magnetic Resonance (MR) images features, MRI texture features, and the possible relationship of MR images features and MRI texture features to evaluate their abilities in contrastive analysis between craniopharyngioma and meningioma.

## 2. Method

### 2.1. Patient Selection

We retrospectively searched our institution database to identify all qualified patients. Eligibility criteria for qualified patients were (1) histopathological confirmation; (2) elaborate electrical medical records; (3) diagnostic MR scan before the operation; and (4) tumors in the sellar/parasellar area. Exclusion criteria were (1) history of treatments before the MR scan; (2) history of intracranial disease (e.g., brain trauma, intracranial infection, or other types of brain tumor); and (3) patients with a recurrent brain tumor considering the interference of scar tissue. Finally, 127 qualified patients with craniopharyngioma (*n* = 63) or meningioma (*n* = 64) were included in this study. All patients underwent surgical resection of tumor in our neurosurgery department from 2014 to 2018.

### 2.2. MRI Protocol

MRI was available in all patients, including contrast-enhanced T1-weighted sequences and T2-weighted sequences. The MR device used is the 3.0 T Siemens Trio Scanner. Contrast-enhanced T1-weighted imaging used gadopentetate dimeglumine (0.1 mmol/Kg) as the contrast agent, acquiring axial, coronal, and sagittal data. The scanning of dynamic enhanced MRI was conducted within 250 s after injection of the contrast agent. Among the 127 patients enrolled in this study, the contrast-enhanced images were available in all patients, while images of T2-weighted imaging (T2WI) were available among 114 patients.

### 2.3. MR Images Features Analysis and Texture Analysis

Two neurosurgeons reviewed all MRI scans to extract qualitative MR images features under the supervision of a senior radiologist and a senior neurosurgeon, with whom disagreements were solved by discussion and consultation. We evaluated the following qualitative MR images features based on the clinical experience and the previous studies: (1) signal intensity compared with normal tissue on contrast-enhanced images and images of T2WI, (2) heterogeneity on contrast-enhanced images and images of T2WI, (3) unenhanced area (s), (4) cystic alteration (s) on contrast-enhanced images or images of T2WI, and (5) the presence of air-fluid level. Besides, the size and the location of tumor tissue were also measured and recorded [3, 18].

LifeX is medical software which reads medical images locally and characterizes tumor heterogeneity. Two neurosurgeons utilized LifeX package (http://www.lifexsoft.org) to extract texture features followed by editing by a senior radiologist and a senior neurosurgeon. ROI was manually drawn along the lesions on contrast-enhanced images or images of T2WI to obtain texture features. Forty-six features were extracted from MR images, including the histogram-based matrix (HISTO), grey-level co-occurrence matrix (GLCM), grey-level run length matrix (GLRLM), grey-level zone length matrix (GLZLM), and neighborhood grey-level dependence matrix (NGLDM). In this study, we performed statistical analyses on 10 most popular and relevant texture features from two matrixes (Energy, Entropy, Kurtosis, and Skewness from HISTO; Contrast, Dissimilarity, Energy, Entropy, and Homogeneity from GLCM). According to previous studies, these texture features are most popular and are of most significance [16, 19]. The explanation of the selected features is provided in Supplementary [Supplementary-material supplementary-material-1].

### 2.4. Statistical Analysis

All statistical analyses were conducted using IBM SPSS Statistics for Windows, Version 22.0 (IBM Corp. Armonk, NY, USA) and MedCalc statistics (MedCalc Software bvba, Acacialaan, Belgium). In statistics processing, we summarized variables based on their classification, the continuous with means and ranges, while the categorical with frequencies and percentages. For the clinical, radiological, and histopathological features, the significant difference between meningioma and craniopharyngioma was examined first with chi-square tests (for categorical variables with enough statistics), Fisher exact tests (for categorical variables with limited statistics), and the Mann–Whitney *U* test (for continuous variables).

As for the texture features, the Mann–Whitney *U* test with the Benjamini–Hochberg method was conducted first to determine if there were significant differences between meningioma and craniopharyngioma, and binary logistic regression analysis was conducted subsequently to predict the probability as independent predictors. Their practical diagnostic value was evaluated with the receiver operating characteristic curve (ROC), from where we got the area under the curve (AUC), standard error, 95% confidence interval (CI), optimal cutoff point value (considered optimal at maximal Youden's index), sensitivity, and specificity.

Finally, the texture features were analyzed with the Mann–Whitney *U* test and ROC analyses successively to investigate the associations between texture features and cystic alteration.

## 3. Result

### 3.1. Patient Selection

The characteristics of patients and lesions are summarized in Table 1. There was predominance in females in meningioma, while there were no significant gender differences in craniopharyngioma, which was in accordance with previous population-based studies [20–22]. As reported before, the bimodal age distribution is observed in craniopharyngioma patients, and adults were the majority of meningioma patients [5]. The average size of craniopharyngioma tumor tissue was approximately larger than meningioma by 8.45 mm. A “Dural tail” adjacent to the tumor was observed in the majority of meningioma while no “Dural tail” was observed in craniopharyngioma.

### 3.2. Qualitative MRI Features Analysis

**Figure 1:**
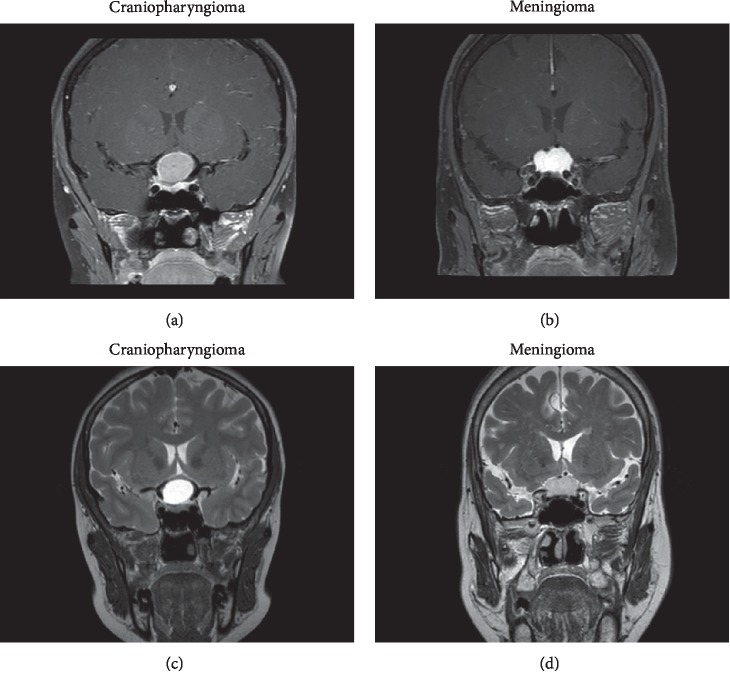
Examples of two cases from the MR images in patients with craniopharyngioma and meningioma. (a) Contrast-enhanced images with craniopharyngioma, (b) a contrast-enhanced image with meningioma, (c) images of T2WI with craniopharyngioma, and (d) an image of T2WI with meningioma.

Among the seven MR images features we analyzed, four of them were found to be significantly different between craniopharyngioma and meningioma (the *p* values of the four MR images features were all less than 0.001). The unenhanced area on contrasted images, hyperintense or extreme hyperintense on images of T2WI, heterogeneity on images of T2WI, and cystic alteration were more likely to be observed in craniopharyngioma. However, the other features, including signal intensity on contrasted images, heterogeneity on contrasted images, and air-fluid level, did not distinguish significantly between the two types of tumors. The details of qualitative MR imaging features analysis are summarized in Table 2. Examples of two cases from the MR images of patients with craniopharyngioma and meningioma are presented in Figure 1.

### 3.3. Quantitative MRI Texture Features Analysis

**Figure 2:**
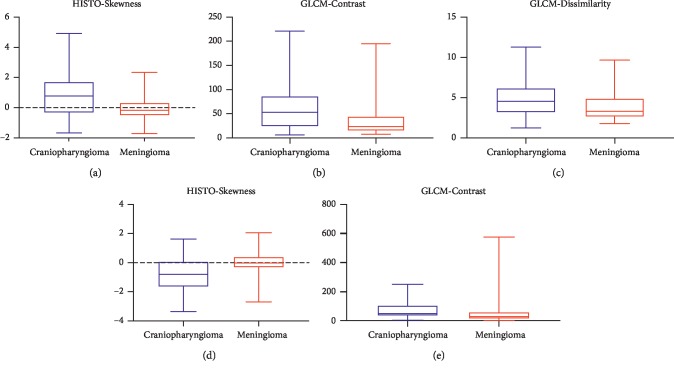
Boxplot of five independent texture features: (a) HISTO-Skewness, (b) GLCM-Contrast, and (c) GLCM-Dissimilarity on contrast-enhanced images; (d) HISTO-Skewness and (e) GLCM-Contrast on images of T2WI in discriminating craniopharyngioma and meningioma. Craniopharyngioma showed higher HISTO-Skewness, GLCM-Contrast, GLCM-Dissimilarity on contrast-enhanced images, and GLCM-Contrast on images of T2WI, but lower HISTO-Skewness on images of T2WI than craniopharyngioma.

According to the Mann–Whitney *U* test, significant differences were observed in five features, including HISTO-Skewness, GLCM-Contrast, GLCM-Dissimilarity from contrast-enhanced images, and HISTO-Skewness, GLCM-Contrast from images of T2WI (the *p* values of the five features are all less than 0.01, the Benjamini–Hochberg correction adjusted level of significance *p*^*∗*^=0.01 considering the variables included in binary logistic regression). Boxplot of five independent texture features is presented in Figure 2. In logistic regression analysis, the collinearity between features was examined first to avoid the interference, and then the statistics were standardized. The results of regression suggested that the HISTO-Skewness and GLCM-Contrast on contrast-enhanced images and the HISTO-Skewness on images of T2WI could be regarded as independent predictors. The outcomes (*p* values, odds ratio (OR), and 95% CI) of the binary regression are presented in Table 3.

**Figure 3:**
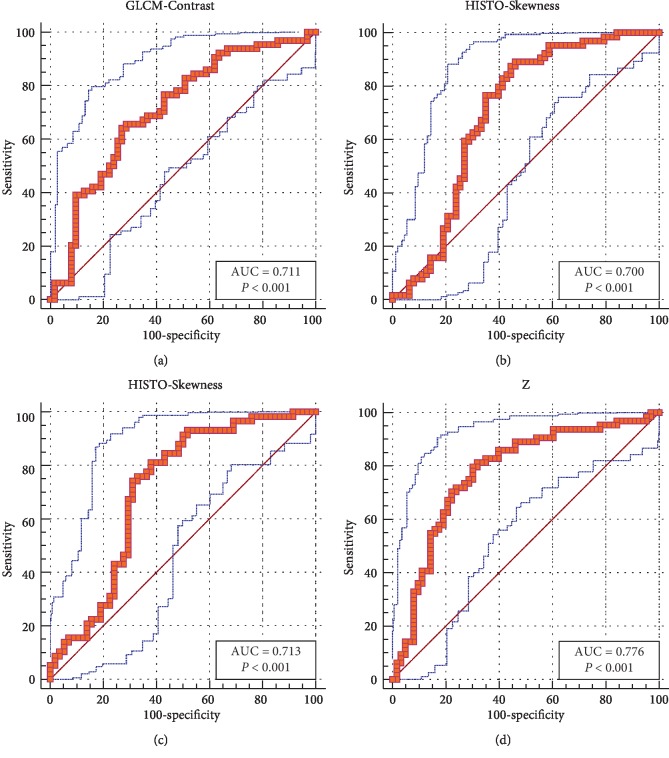
Receiver operating characteristic (ROC) curves of (a) GLCM-Contrast, (b) HISTO-Skewness on contrast-enhanced images, and (c) HISTO-Skewness on images of T2WI demonstrated promising diagnostic value of the three texture features, of which area under curves (AUC) were all more than 0.700. (d) ROC curves of an integrated model combining GLCM-Contrast and HISTO-Skewness on contrast-enhanced images showed more value in practical diagnosis with higher AUC.

ROC curves were only performed in three independent predictors (HISTO-Skewness, GLCM-Contrast on contrast-enhanced images, and the HISTO-Skewness on T2WI). AUC of these texture features were all higher than 0.700, which presented their practical value in contrastive analysis. The outcomes are presented in Figure 3 and Table 4.

Considering the diagnostic value of a single texture feature was not good enough to be taken as a practical parameter, the integrated model was built based on the results of binary logistic regression. The formula of the model is

(1) $$\begin{array}{c} z\text{ score}=\left(-0.892*\text{HISTOskewness}\right)-\left(2.438*\text{GLCMcontrast}\right). \end{array}$$

The ROC curve shown in Figure 3 showed the AUC of the integrated model was 0.776, representing higher diagnostic value than any single texture feature.

### 3.4. The Relationship between MRI Images Features and MRI Texture Features

**Figure 4:**
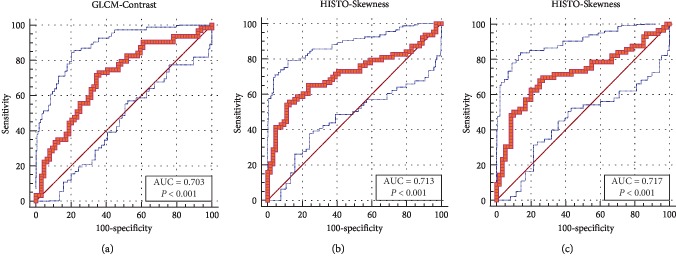
Receiver operating characteristic (ROC) curves of (a) GLCM-Contrast, (b) HISTO-Skewness on contrast-enhanced images, and (c) HISTO-Skewness on images of T2WI demonstrated MR images features and texture features were related to each other.

According to the Mann–Whitney *U* test, significant differences were observed in three features: HISTO-Skewness, GLCM-Contrast on contrast-enhanced images, and HISTO-Skewness on images of T2WI (the *p* values of the three MR texture features were all less than 0.05). The results of ROC analysis suggested HISTO-Skewness, GLCM-Contrast on contrast-enhanced images, and HISTO-Skewness on images of T2WI were statistically significant. The outcomes of ROC analysis are presented in Figure 4.

## 4. Discussion

Clinically, there still remained to be a challenge in the contrastive analysis of craniopharyngioma and meningioma in the sellar/parasellar area because craniopharyngiomas may radiologically mimic meningiomas [12, 23]. In this study, we investigated the abilities of MRI scan traits to facilitate contrastive analysis between craniopharyngioma and meningioma in both MR images features and texture features. Besides, we also evaluated the relationship between MR images features and texture features, so that we can combine them to improve the accuracy of discrimination. To the best of our knowledge, our study was the first study to combine the two features together.

Previous studies had shown us a lot about the MR images features of craniopharyngioma and meningioma. Craniopharyngioma is typically a solid-cystic, lobular tumor with calcareous concretions of the intra- and/or suprasellar region, [24–26]. Clinically, MRI was not implemented to characterize calcifications because of the poor performance in discriminating calcareous concretions from neighbor tissues, which can be definitively detected or excluded with computerized tomography [3]. Meningiomas present isointense on images of T1WI and T2WI with typically a strong homogeneous enhancement following administration of gadolinium contrast as a result of the absence of a blood-brain barrier [12, 18, 27]. Besides, a linear, enhancing dural tail extending away from the tumor tissue was in the majorities [28, 29]. In our study, we took seven MR images features into analysis, and the results demonstrated cystic alteration (s), unenhanced area (s), unenhanced area (s), and heterogeneity on images of T2WI were significantly different between them, which was in accordance with previous studies. During the MRI features extracting process, subjectivity was inevitable, even though the whole process was under the supervision of the senior radiologist and the senior neurosurgeon. The protein concentration within the cystic fluid can contribute to the variation of signal intensity on MR images of craniopharyngioma, and the standard of the signal intensity is relatively subjective. Furthermore, some craniopharyngioma represented similar characters to meningioma. Based on these facts, texture analysis can be implemented as a more accurate and objective method [3, 23].

Texture analysis has been applied to improving the accuracy in classifying and grading meningioma, but texture analysis on craniopharyngioma has not been reported yet [17, 30, 31]. Texture analysis was reported as a potential, noninvasive tool to reflect tumor heterogeneity in recent research studies. It was able to improve the accuracy of classification, grade of tumors, and differential diagnosis between tumors [32]. Considering that the assessment of images feature was relatively subjected, in our study, we took ten texture features derived from two matrixes into consideration which are most studied [16, 19]. Four of them were derived from the histogram-based matrix (HISTO, the first-order statistics), while the others were derived from the grey-level co-occurrence matrix (GLCM, second-order statistics). The histogram-based matrix only described the frequency distribution of voxel values, disregarding the inherent spatial relationship among voxel values, while the GLCM matrix, on the other hand, accounted for the spatial voxel-values organization. Therefore, we took these texture features derived from HISTO and GLCM into consideration, so that we could assess the MR images in the overall view and partial view. HISTO-Skewness and GLCM-Contrast on contrast-enhanced images and HISTO-Skewness on images of T2WI represented promising abilities in contrastive analysis. There were significant differences in them, with all the *p* values less than 0.001, which means the frequency distribution of voxel values and the spatial voxel-values organization is significantly discriminated between craniopharyngioma and meningioma [32]. The binary logistic regression demonstrated that HISTO-Skewness and GLCM-Contrast on contrast-enhanced images and HISTO-Skewness on images of T2WI could be useful as independent diagnostic factors. However, each single texture feature was not good enough to be taken as a practical parameter because of their limited diagnostic value. Therefore, we built an integrated model to take the probable relationship between HISTO-Skewness and GLCM-Contrast into consideration, which received an AUC higher than that of each single texture feature.

Previous studies determined that craniopharyngioma is typically a cystic tumor and the cystic alteration could be regarded as the statistical demarcations between craniopharyngioma and meningioma. Meanwhile, variant protein concentration within the cystic fluid could result in variant signal intensity in MRI. Thus, we carried out an analyzation to detect the possible relationship between texture features and cystic alteration [16]. HISTO-Skewness and GLCM-Contrast on contrast-enhanced images and HISTO-Skewness on images of T2WI showed promising results (all the *p* values were less than 0.001). Previous studies have reported the high relationships between histopathology and MR images features. These studies demonstrated that the differences in histopathology can present variant intensity in MR images, for instance, the immobile bloodstream and inflow effect of the tumor could result in high T2WI signal [33] A previous study also demonstrated the capability of texture features in detecting these histopathological lesions. Theoretically, there were strong relationships between MR images features and texture features, and to some extent, they both related to the histopathologic features. The MR images features characterized tumors in the macroview, while the texture features did it in the microview. There being no studies on the relationship before, we attempted to demonstrate it, and the results implied that MR images features and texture features were related to each other.

Our study had several limitations. First, as a retrospective study, we only included patients with surgically resectable tumors. Second, the potential for selection biases could not be excluded. Third, we were unable to assess other subsequences, especially conventional T1WI. Fourth, the differences in tumor subtypes were not taken into consideration because of the limited number of patients.

## 5. Conclusions

MR images features (cystic alteration) and texture features (HISTO-Skewness and GLCM-Contrast on contrast-enhanced images and HISTO-Skewness on images of T2WI) were useful in the contrastive analysis between craniopharyngioma and meningioma. Besides, the two types of features were related to each other. But, more studies are required to verify our results and rectify the defects.

## Figures and Tables

**Table 1:** Characteristics of the patient and lesion.

Character	Craniopharyngioma	Meningioma
Gender	Male: 37 (58.7)	Male: 18 (28.1)
	Female: 26 (41.3)	Female: 46 (71.9)
Age (years)	31.62 (2∼73)	49.19 (9∼72)
Tumor size (mean ± SD (mm))	28.86 ± 9.57	20.41 ± 5.96
Location	Intrasellar: 0	Intrasellar: 0
	Intra- and suprasellar: 17	Intra- and suprasellar: 8
	Suprasellar: 46	Suprasellar: 56
Dural tail sign	0	62

**Table 2:** The differences in MR images features between craniopharyngioma and meningioma. Entries in bold were significant.

Qualitative MR features		Craniopharyngioma *N* (%)	Meningioma *N* (%)	*p* value
Signal intensity on contrasted images	Hypointense	0 (0)	2 (3)	0.149
	Isointense	0 (0)	0 (0)	
	Hyperintense	40 (63)	32 (50)	
	Extreme hyperintense	23 (37)	30 (47)	
Heterogeneity on contrasted images	Homogenous	7 (11)	7 (11)	0.975
	Heterogeneous	56 (89)	57 (89)	
Unenhanced area (s)	Presence	50 (79)	6 (9)	**<0.001**
	Absence	13 (21)	58 (91)	
Signal intensity on T2WI	Hypointense	0 (0)	1 (2)	**<0.001**
	Isointense	6 (10)	46 (81)	
	Hyperintense	14 (25)	10 (17)	
	Extreme hyperintense	37 (65)	0 (0)	
Heterogeneity on T2WI	Homogenous	10 (18)	39 (68)	**<0.001**
	Heterogeneous	47 (82)	18 (32)	
Cystic alteration (s)	Presence	58 (92)	5 (8)	**<0.001**
	Absence	5 (8)	59 (92)	
Air-fluid level	Presence	7 (11)	0 (0)	0.006
	Absence	56 (89)	64 (1)	

T2WI: T2-weighted imaging.

**Table 3:** The binary logistic regression on texture features between craniopharyngioma and meningioma.

Texture feature		*p* value	OR	95% CI
Contrast-enhanced images on T1WI	HISTO-skewness	**0.001**	0.410	0.242–0.693
	GLCM-contrast	**0.037**	0.087	0.009–0.863
	GLCM-dissimilarity	0.145	4.637	0.588–36.560
Images of T2WI	HISTO-skewness	**<0.001**	2.458	1.534–3.940
	GLCM-contrast	0.086	0.635	0.378–1.066

Entries in bold were significant. HISTO: histogram-based matrix, GCLM: grey-level co-occurrence matrix, T1WI: T1-weighted imaging, T2WI: T2-weighted imaging, OR: odds ratio, CI: confidence interval.

**Table 4:** Diagnostic performance of texture features for differentiating craniopharyngioma from meningioma.

Texture parameter		AUC	Standard error	95% CI	Cutoff point	Sensitivity	Specificity
Contrast-enhanced images on T1WI	HISTO-Skewness	0.700	0.0491	0.612∼0.778	0.648	87.50	55.56
	GLCM-Contrast	0.711	0.046	0.624∼0.788	29.444	64.06	73.02
	*Z*-score	0.776	0.043	0.693∼0.845	0.093	79.69	69.84
Images of T2WI	HISTO-Skewness	0.713	0.050	0.612∼0.793	−0.308	74.14	68.97

HISTO: histogram-based matrix, GCLM: grey-level co-occurrence matrix, T1WI: T1-weighted imaging, T2WI: T2-weighted imaging, AUC: area under the curve, CI: confidence interval.

## Data Availability

The data used to support the findings of this study are available from the corresponding author upon request.

## Supplementary Materials

Supplementary Materials Supplementary Table 1: the explanation of the selected features. Abbreviations: HISTO: histogram-based matrix; GCLM: grey-level co-occurrence matrix.
