# Supplementary material for: Radiomic Analysis of Craniopharyngioma and Meningioma in the Sellar/Parasellar Area with MR Images Features and Texture Features: A Feasible Study
Source: Contrast Media Mol Imaging. 2020 Feb 18;2020:4837156. doi: 10.1155/2020/4837156 (PMC7049426; doi:10.1155/2020/4837156)
Supplement: Supplementary Materials — Supplementary Table 1: the explanation of the selected features. Abbreviations: HISTO: histogram-based matrix; GCLM: grey-level co-occurrence matrix. [file 4837156.f1.docx]

**The explanation of the selected features**

| Parameters | | Explanation |
| --- | --- | --- |
| Parameters from Histogram | | |
|  | HISTO_Skewness | the asymmetry of the grey-level distribution in the histogram |
|  | HISTO_Kurtosis | the shape of the grey-level distribution relative to a normal distribution |
|  | HISTO_Entropy | the randomness of the distribution |
|  | HISTO_Energy | the uniformity of the distribution |
| Parameters from grey level co-occurrence matrix (GLCM) | | |
|  | GLCM_Homogeneity | the homogeneity of grey-level voxel pairs |
|  | GLCM_Energy | the uniformity of grey-level voxel pairs |
|  | GLCM_Contrast | the local variations in the GLCM |
|  | GLCM_Correlation | the local variations in the GLCM |
|  | GLCM_Entropy | the local variations in the GLCM |
|  | GLCM_Dissimilarity | the local variations in the GLCM |
